# Translating Anti-Inflammatory Strategies for Atherosclerosis: Deep Phenotyping, Next-Generation Drug Targets, and Precision Medicine

**DOI:** 10.3390/cells13151306

**Published:** 2024-08-05

**Authors:** Yaw Asare, Marios K. Georgakis

**Affiliations:** 1 Institute for Stroke and Dementia Research, Ludwig-Maximilians-University (LMU) Hospital, LMU Munich, 81377 Munich, Germany; 2 Program in Medical and Population Genetics, Broad Institute of MIT and Harvard, Cambridge, MA 02142, USA

Atherosclerosis is the main pathology underlying cardiovascular disease (CVD), including myocardial infarction and ischemic stroke. Atherosclerosis arises as a result of maladaptive inflammatory responses to subendothelial lipoprotein particle accumulation in medium- and large-sized arteries [1]. Despite the success of lipid-lowering medications in mitigating the complications of atherosclerosis, CVD remains the main cause of death and adult disability worldwide [2]. Hence, the development of novel preventive and therapeutic strategies is necessary to alleviate the global burden of CVD. Treatments targeting the immune landscape of atherosclerosis have emerged as a promising new pillar of atheroprotective approaches.

A role of inflammation in atherosclerosis had long been suggested. Histological studies of human and animal atherosclerosis lesions revealed immune cell infiltration within atherosclerotic plaques [3]. Furthermore, animal studies intervening with immune mechanisms showed changes in the course of atherogenesis in experimental models of atherosclerosis [4]. Epidemiological studies further found associations of inflammatory biomarkers with CVD risk [5]. Only recently, though, have large clinical trials provided proof-of-concept evidence that targeting inflammation can lower CVD risk, thus setting the stage for a new paradigm of atheroprotective treatments [6,7,8]. Colchicine, tested in COLCOT and LoDoCo2, has recently received FDA approval as the first anti-inflammatory drug for the treatment of atherosclerotic CVD [9].

However, anti-inflammatory approaches have not been widely implemented in the clinic due to associations with higher risk of infections, a lack of understanding of the immune mechanisms driving atheroprogression, and the very high interindividual variability in drug response. Current research efforts are focused on (1) a deeper immunophenotyping of atherosclerosis (2), a better understanding of the precise immune mechanisms that promote atheroprogression, and (3) the selection of the patients that might benefit from atheroprotective treatments. In this Special Issue on “Translating Anti-Inflammatory Strategies for Atherosclerosis: Deep Phenotyping, Next-Generation Drug Targets, and Precision Medicine”, specific aspects of these three axes are discussed. 

Deeper phenotyping with novel technologies, such as single-cell transcriptomics, offers unique insights into the complexity of the immune microenvironment in human atherosclerosis. These studies have offered a new window into the subtypes of known immune cell players in atherosclerosis, such as macrophages, and have provided novel insights into the role of underappreciated cells, such as T cells. Blagov et al. summarize their insights into the different macrophage subtypes, focusing on the dichotomy between pro-inflammatory M1 macrophages that promote atheroprogression and anti-inflammatory M2 macrophages that promote atheroprogression (Contribution 1). Macrophages may create a pro- or anti-inflammatory atherosclerosis microenvironment that might promote their transformation to foam cells or the stabilization of atherosclerotic lesions. We have only recently started to understand the molecular mechanisms underlying the phenotypic shift between the key macrophage subtypes. Hinkley et al. focus on T cells, the role of which was underappreciated until recently (Contribution 2). They discuss the activation and polarization of T cells into pro-inflammatory subsets, such as Th1 and Th17 cells that crucially contribute to chronic atheroinflammation, as well as the balance with regulatory T cell subsets. Understanding the complex immune landscape of human atherosclerosis can enable the optimization of drug target selection for future treatments, as well as providing an explanation for the highly heterogeneous fate of different lesions.

Dissecting the role of druggable immune mechanisms in atheroprogression remains a key challenge, and several molecular targets including endogenous tissue inhibitors are under investigation to assess their therapeutic potential for atheroprotection. The endogenous inhibitor of metalloproteinases TIMP-1 is traditionally recognized for its role in regulating matrix metalloproteinases (MMPs), enzymes pivotal in extracellular matrix remodeling. However, the findings from Ebert and colleagues (Contribution 3) now suggest that TIMP-1 has facets beyond its conventional role, revealing its interaction with CD74 through shared residues with MIF and MIF-2. Having identified an interaction between TIMP-1 and CD74, they next performed a phospho-kinase array and found TIMP-1-dependent activation in the serine/threonine kinase AKT and ERK1/2 in human monocytes. These pathways are crucial for cell survival, proliferation, and migration and thus hint at the broader impact of TIMP-1 on monocyte function. Indeed, their functional assays provided compelling evidence for a TIMP-1–CD74 axis in the recruitment of human monocytes and the proliferation of VSMC. To contextualize these experimental findings within human pathology, they re-analyzed scRNA-seq data from human atherosclerotic lesions and found high expression of TIMP-1 and CD74 in monocytes, macrophages, and VSMCs. These data, in conjunction with the experimental evidence, underscore the relevance of the TIMP-1–CD74 axis in the inflammatory milieu of atherosclerosis. This ability of TIMP-1 to modulate monocyte recruitment and VSMC proliferation through CD74 engagement suggests a potential role in vascular inflammation and atherosclerosis but remains to be tested in more complex preclinical disease models.

To highlight the complexity and dominant role of innate immune mechanisms driving atheroprogression, Aronova and colleagues summarize our understanding of the key pathways driving chronic inflammation in atherosclerosis and further describe how they may operate together to dictate the reprogramming of immune cells (Contribution 4). They highlight innate immune signaling cascades, including scavenger receptor-mediated pathways, TLR signaling, and inflammasome activation, that have emerged as important regulators of chronic inflammation in atheroprogression, the inhibition of which may provide a vital tool in reducing vascular inflammation. They further describe how the recent emergence of new technologies, including genetic lineage tracing and fate mapping, CyTOF, scRNA-seq, and spatial transcriptomics, has helped in the deep phenotyping of atherosclerosis. As the search for a second generation of anti-inflammatory strategies continues, unraveling the cellular and molecular mechanisms governing atheroprogression presents a unique opportunity for new classes of therapeutics for atherosclerosis.

To be successful in designing targeted therapies for CVD prevention, we may be required to take a holistic approach to understanding the underlying mechanisms of CVD. To this end, Mohanta and colleagues propose a new concept, the “Neuroimmune Cardiovascular Circuit Hypothesis”, for CVD pathogenesis (Contribution 5). They describe how three interconnected biological systems—the nervous, immune, and cardiovascular systems—form a responsive network that regulates cardiovascular function in health and disease. They further highlight how within the cardiovascular system, two sub-circuits, the artery brain circuit and the heart brain circuit, form a larger cardiovascular brain circuit. Having addressed how the ABC and HBC form a larger CBC, they next summarize how the immune system, comprising innate and adaptive immunity, interacts with the CBC and the nervous system. Collectively, their review integrates recent cardiovascular neurobiology findings into existing cardiovascular disease models. An understanding of the cardiovascular brain circuit could lead to new therapies for CVD, as emerging biologically active molecules are identified.

The detection of novel biomarkers of atheroinflammation is a major aspect of the process of translating immunotherapies to the clinic. As illustrated in CANTOS [6], there is substantial interindividual variability in the benefit of the treatment. Biomarkers of atheroinflammation will enable (1) the optimal selection of patients that might benefit from specific treatments and (2) the monitoring of treatment response or risk change over time as a readout. In this context, McCabe and colleagues discuss PET imaging as a modality for quantifying atherosclerotic plaque inflammation in the carotid arteries (Contribution 6). 18F-fluorodeoxyglucose (FDG) is taken up by metabolically active macrophages and accumulates at tissue sites with increased inflammatory activity. Imaging–histology correlation studies in patients undergoing carotid endarterectomy have shown a robust association between macrophage content in the excised plaque and pre-surgery FDG uptake in the carotid plaques. More recently, prospective studies have further provided evidence that FDG uptake in carotid PET imaging is associated with future risk of major adverse cardiovascular events and ischemic stroke. FDG-PET uptake has been used as a surrogate endpoint of efficacy in phase 2 clinical trials primarily testing statins, showing significant changes in short-term follow-ups of 3 months. Beyond FDG, a number of novel tracers have been developed that appear to be more specific to arterial inflammation and to exert more favorable signal-to-noise ratios. The authors provide a meticulous discussion of current limitations, especially with regard to the standardization of image acquisition and analysis, the exposure to radiation, and the practicality of PET imaging in cardiovascular prevention.

Exciting new technologies are expected to advance our understanding of the immunology of atherosclerosis and contribute to the development of personalized therapies (Figure 1). Firstly, deeper phenotyping, utilizing cutting-edge omics technologies, such as single-cell and spatial transcriptomics, will be essential in elucidating the complex immune landscape of human atherosclerosis [10]. Our current models of atheroprogression, which define endophenotypes for experimental, translational, and clinical research, are entirely based on histopathology and do not consider the immune microenvironment of the lesions. Secondly, incorporating multi-omics data could provide key insights into the discovery of a next generation of drug targets for atheroprotective immunotherapies that will overcome the limitations of tested approaches, such as a high risk of infections [11,12,13]. A deeper characterization of potential druggable mechanisms in follow-up in vitro experiments or animal models of atherosclerosis will be key in allowing them to progress to clinical development. Finally, the development and validation of novel biomarkers for atheroinflammation will be crucial in selecting the patients most likely to benefit from specific treatments and monitoring the therapeutic efficacy. Already, advances in imaging techniques such as vascular MRI, CT angiography, or PET utilizing specific radiotracers are enabling a personalized assessment of vascular risk [14]. Omics analyses in peripheral blood, such as metabolomics and proteomics, could allow the detection of signatures of atheroinflammation that are better surrogates than the existing non-specific biomarkers, such as high-sensitivity C-reactive protein.

## Figures and Tables

**Figure 1:**
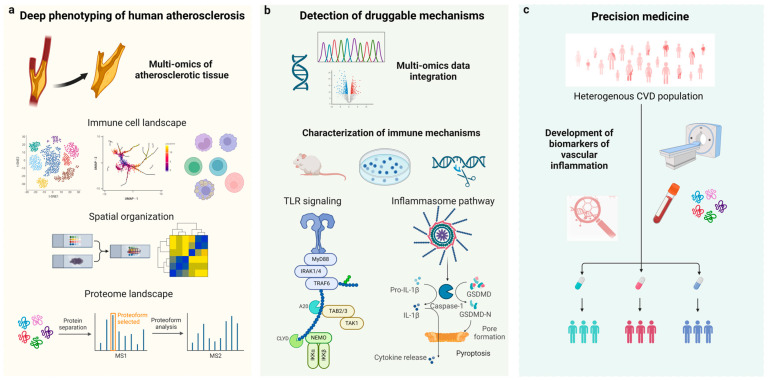
Translating anti-inflammatory strategies for atherosclerosis. (**a**) The recent emergence of new technologies including scRNA-seq, ATAC-seq, mass cytometry, and spatial omics presents unique opportunities for the deep phenotyping of atherosclerotic plaques beyond traditional immunohistochemistry techniques. (**b**) The integration of these large-scale datasets will allow the detection of druggable mechanisms and molecular targets that can be scrutinized further in experimental models to assess causality and elucidate pathways with central roles in the pathogenesis of atherosclerosis. This will aid in the identification of molecular targets for therapeutic intervention in atherosclerotic cardiovascular disease. (**c**) The development and validation of biomarkers for atheroinflammation will be crucial in selecting the patients most likely to benefit from specific treatments and in monitoring therapeutic efficacy.

## References

[1] Björkegren J.L., Lusis A.J. (2022). Atherosclerosis: Recent developments. Cell.

[2] Martin S.S., Aday A.W., Almarzooq Z.I., Anderson C.A., Arora P., Avery C.L., Baker-Smith C.M., Gibbs B.B., Beaton A.Z., Boehme A.K. (2024). 2024 Heart Disease and Stroke Statistics: A Report of US and Global Data From the American Heart Association. Circulation.

[3] Aqel N.M., Ball R.Y., Waldmann H., Mitchinson M.J. (1985). Identification of macrophages and smooth muscle cells in human ath-erosclerosis using monoclonal antibodies. J. Pathol..

[4] Smith J.D., Trogan E., Ginsberg M., Grigaux C., Tian J., Miyata M. (1995). Decreased atherosclerosis in mice deficient in both mac-rophage colony-stimulating factor (op) and apolipoprotein E. Proc. Natl. Acad. Sci. USA.

[5] Ridker P.M., Cushman M., Stampfer M.J., Tracy R.P., Hennekens C.H. (1997). Inflammation, aspirin, and the risk of cardiovascular disease in apparently healthy men. N. Engl. J. Med..

[6] Ridker P.M., Everett B.M., Thuren T., MacFadyen J.G., Chang W.H., Ballantyne C., Fonseca F., Nicolau J., Koenig W., Anker S.D. (2017). Antiinflammatory Therapy with Canakinumab for Atherosclerotic Disease. N. Engl. J. Med..

[7] Tardif J.-C., Kouz S., Waters D.D., Bertrand O.F., Diaz R., Maggioni A.P., Pinto F.J., Ibrahim R., Gamra H., Kiwan G.S. (2019). Efficacy and Safety of Low-Dose Colchicine after Myocardial Infarction. N. Engl. J. Med..

[8] Nidorf S.M., Fiolet A.T.L., Mosterd A., Eikelboom J.W., Schut A., Opstal T.S.J., Salem H.K., Fu X., Ireland M.A., Lenderink T. (2020). Colchicine in Patients with Chronic Coronary Disease. N. Engl. J. Med..

[9] Buckley L.F., Libby P. (2024). Colchicine’s Role in Cardiovascular Disease Management. Arter. Thromb. Vasc. Biol..

[10] Fernandez D.M., Giannarelli C. (2022). Immune cell profiling in atherosclerosis: Role in research and precision medicine. Nat. Rev. Cardiol..

[11] Georgakis M.K., Bernhagen J., Heitman L.H., Weber C., Dichgans M. (2022). Targeting the CCL2–CCR2 axis for atheroprotection. Eur. Hear. J..

[12] Gill D., Georgakis M.K., Walker V.M., Schmidt A.F., Gkatzionis A., Freitag D.F., Finan C., Hingorani A.D., Howson J.M.M., Burgess S. (2021). Mendelian randomization for studying the effects of perturbing drug targets. Wellcome Open Res..

[13] Prapiadou S., Živković L., Thorand B., George M.J., van der Laan S.W., Malik R., Herder C., Koenig W., Ueland T., Kleveland O. (2024). Proteogenomic Data Integration Reveals CXCL10 as a Potentially Downstream Causal Mediator for IL-6 Signaling on Atherosclerosis. Circulation.

[14] Antonopoulos A.S., Angelopoulos A., Papanikolaou P., Simantiris S., Oikonomou E.K., Vamvakaris K., Koumpoura A., Farmaki M., Trivellam M., Vlachopoulos C. (2022). Biomarkers of Vascular Inflammation for Cardiovascular Risk Prognostication: A Meta-Analysis. JACC Cardiovasc. Imaging.

